# Supplementary material for: PKCiota Inhibits the Ferroptosis of Esophageal Cancer Cells via Suppressing USP14-Mediated Autophagic Degradation of GPX4
Source: Antioxidants (Basel). 2024 Jan 17;13(1):114. doi: 10.3390/antiox13010114 (PMC10812620; doi:10.3390/antiox13010114)
Supplement: Supplementary file 1 [file antioxidants-13-00114-s001.zip › Supplement figure legends.pdf]

Supplement figure legends

Figure S1. RSL3 treatment decreased the mRNA level of PKCiota in KYSE510 and KYSE450 cells. **, $p < 0.01$; ***, $p < 0.001$.

Figure S2. FIN56 treatment decreased the protein level of PKCiota but Erastin treatment had no effect on the protein level of PKCiota in KYSE510 and KYSE450 cells. 0, 10, 20 and 40 μM of FIN56 and 0, 20, 40 and 80 μM of Erastin were used to treat the ESCC cells.

Figure S3. Silence PKCiota had no effect on the mRNA level of GPX4 in KYSE510 and KYSE450 cells. ns, not significant.

Figure S4. miR-145-5p was decreased in ESCC. Transcript levels of miR-145-5p in normal tissues and ESCC tissues from GSE43732, GSE114110 and GSE145198. *, $p < 0.05$; ****, $p < 0.0001$.
