# Supplementary figures and images for: PKCiota Inhibits the Ferroptosis of Esophageal Cancer Cells via Suppressing USP14-Mediated Autophagic Degradation of GPX4
Source: Antioxidants (Basel). 2024 Jan 17;13(1):114. doi: 10.3390/antiox13010114 (PMC10812620; doi:10.3390/antiox13010114)

Relative expression of PKC $\alpha$  compared with GAPDH

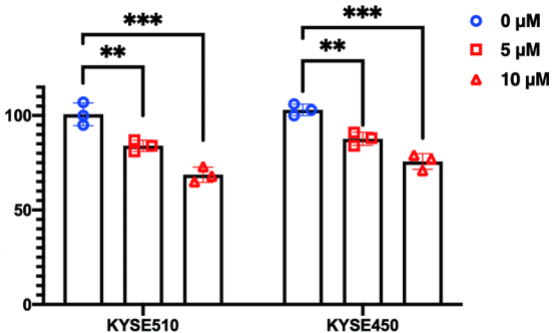

Supplement: Supplementary file 1 [file antioxidants-13-00114-s001.zip › Figure S1.pdf]

**KYSE510**

**KYSE450**

**0 10 20 40**

**0 10 20 40**

**(FIN56,  $\mu$ M)**

**74kDa**

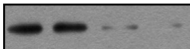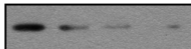

**PKCιota**

**36kDa**

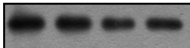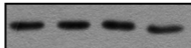

**GAPDH**

**0 20 40 80**

**0 20 40 80**

**(Erastin,  $\mu$ M)**

**74kDa**

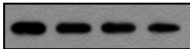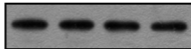

**PKCιota**

**36kDa**

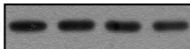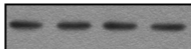

**GAPDH**

Supplement: Supplementary file 1 [file antioxidants-13-00114-s001.zip › Figure S2.pdf]

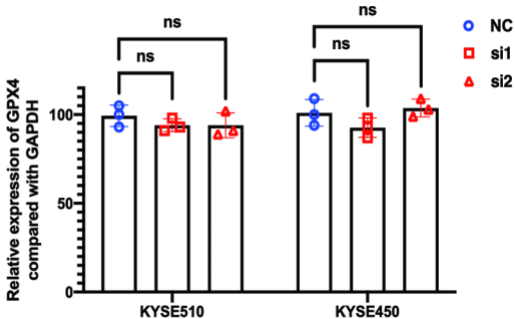

Supplement: Supplementary file 1 [file antioxidants-13-00114-s001.zip › Figure S3.pdf]

**GSE43732**

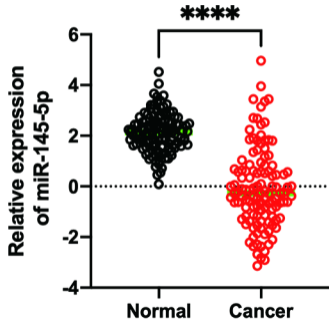

**GSE114110**

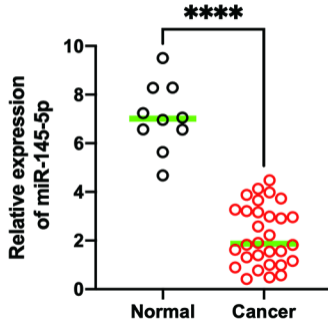

**GSE145198**

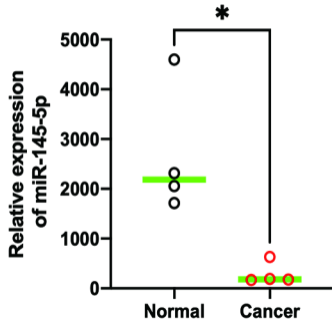

Supplement: Supplementary file 1 [file antioxidants-13-00114-s001.zip › Figure S4.pdf]
